# Phasic Store-Operated Ca^2+^ Entry During Excitation-Contraction Coupling in Skeletal Muscle Fibers From Exercised Mice

**DOI:** 10.3389/fphys.2020.597647

**Published:** 2020-11-12

**Authors:** Elena Lilliu, Karlheinz Hilber, Bradley S. Launikonis, Xaver Koenig

**Affiliations:** ^1^Department of Neurophysiology and Pharmacology, Center for Physiology and Pharmacology, Medical University of Vienna, Vienna, Austria; ^2^School of Biomedical Sciences, The University of Queensland, Brisbane, QLD, Australia

**Keywords:** store-operated Ca^2+^-entry, skeletal muscle, mouse, exercise, electrical field stimulation

## Abstract

Store-operated calcium entry (SOCE) plays a pivotal role in skeletal muscle physiology as, when impaired, the muscle is prone to early fatigue and the development of different myopathies. A chronic mode of slow SOCE activation is carried by stromal interaction molecule 1 (STIM1) and calcium-release activated channel 1 (ORAI1) proteins. A phasic mode of fast SOCE (pSOCE) occurs upon single muscle twitches in synchrony with excitation-contraction coupling, presumably activated by a local and transient depletion at the terminal cisternae of the sarcoplasmic reticulum Ca^2+^-stores. Both SOCE mechanisms are poorly understood. In particular, pSOCE has not been described in detail because the conditions required for its detection in mouse skeletal muscle have not been established to date. Here we report the first measurements of pSOCE in mouse extensor digitorum longus muscle fibers using electrical field stimulation (EFS) in a skinned fiber preparation. We show moderate voluntary wheel running to be a prerequisite to render muscle fibers reasonably susceptible for EFS, and thereby define an experimental paradigm to measure pSOCE in mouse muscle. Continuous monitoring of the physical activity of mice housed in cages equipped with running wheels revealed an optimal training period of 5–6 days, whereby best responsiveness to EFS negatively correlated with running distance and speed. A comparison of pSOCE kinetic data in mouse with those previously derived from rat muscle demonstrated very similar properties and suggests the existence and similar function of pSOCE across mammalian species. The new technique presented herein enables future experiments with genetically modified mouse models to define the molecular entities, presumably STIM1 and ORAI1, and the physiological role of pSOCE in health and under conditions of disease.

## Introduction

Store-operated calcium entry (SOCE) is a widespread cellular mechanism, by which calcium (Ca^2+^) influx across the plasma membrane is triggered by a depletion of the Endo/Sarcoplasmic reticulum (ER/SR) Ca^2+^ stores (Prakriya and Lewis, 2015). Stromal interaction molecule (STIM) acts as the SR Ca^2+^ sensor (Roos et al., 2005; Dirksen, 2009; Prakriya and Lewis, 2015) to orchestrate the activation of Ca^2+^-release activated Ca^2+^ channel (ORAI) in the plasma membrane (Feske et al., 2006; Vig et al., 2006; Dirksen, 2009). STIM1 and ORAI1 are highly expressed in skeletal muscle (Stiber et al., 2008; Vig et al., 2008), and deficiency of either protein abolished SOCE (Lyfenko and Dirksen, 2008). Loss as well as gain of function mutations within STIM1 and ORAI1 affect SOCE and result in the development of skeletal myopathy (Feske et al., 2006; Stiber et al., 2008; McCarl et al., 2009; Böhm et al., 2013, 2014, 2017; Endo et al., 2015).

Store-operated calcium entry is well understood, e.g., in the course of immune cell activation (Prakriya and Lewis, 2015), but its role in skeletal muscle remains elusive. While SOCE is a relatively slow process in non-excitable cells (Launikonis et al., 2010; Trebak et al., 2013) it presents with extraordinary fast kinetics in skeletal muscle (Launikonis and Ríos, 2007; Edwards et al., 2010; Launikonis et al., 2010; Koenig et al., 2018, 2019) suggesting a close juxtaposition or even physical association of STIM1 and ORAI1 within the triads (Dirksen, 2009; Launikonis et al., 2010; Wei-Lapierre et al., 2013; Koenig et al., 2018). Recently, we demonstrated that SOCE is activated upon individual action potentials (APs) in rat skeletal muscle cells (Koenig et al., 2018, 2019), a mode of SOCE that we named phasic SOCE (pSOCE), because it showed comparable kinetics to excitation-contraction coupling.

Action potentials in the transverse tubular system (t-system) of skinned skeletal muscle fibers can be triggered by electrical field stimulation (EFS), and respective muscle twitches are indistinguishable from twitches in intact fibers (Posterino et al., 2000; Posterino and Lamb, 2003). While EFS works well in rat extensor digitorum longus (EDL) muscle fibers, respective experiments presented challenging in mice. The reason for that is unknown, but might relate to the fact that only fibers derived from rats express high enough numbers of voltage-gated sodium channels (VGSCs) within the t-tubular system to enable sufficient membrane depolarization and subsequent activation of the voltage-sensor to trigger Ca^2+^-release from the SR via the ryanodine receptor. Importantly, until now, this has precluded the assessment of pSOCE in mice, and thereby the study of its functional and pathophysiological relevance in skeletal muscle by genetically modified mouse models.

Here we report that moderate physical training of mice by voluntary wheel running for several days enabled successful EFS of skinned mouse EDL fibers in a manner fully comparable to EFS of skinned rat EDL fibers. The EFS- triggered release of SR Ca^2+^ induced an activation of pSOCE, which was indistinguishable from that observed in rat EDL fibers. Our results demonstrate the existence of pSOCE in skeletal muscle fibers across animal species and open the door for future experiments on genetically modified mice to study the role of pSOCE in health and disease.

## Materials and Methods

### Ethics Statement

The current study conforms to the guiding principles of the Declaration of Helsinki and coincides with the rules of the Animal Welfare Committee at the Medical University of Vienna. The current study is covered by the animal ethics vote BMBWF 2020-0.499.046 granted by the Federal Ministry of the Republic of Austria.

### Animal Model and Skinned Fiber Preparation

Male C57BL/6 mice at an age of 10–15 weeks were used throughout this study. Data derived from rat, as presented in Figure 5, are taken from previous work (Koenig et al., 2018), with permission. On the day of experiment animals were killed by cervical dislocation and the EDL muscle was rapidly excised. Isolation of single fibers and mechanical “skinning” of the fibers was performed as previously described (Cully et al., 2016; Koenig et al., 2018, 2019). Briefly, EDL muscle was transferred to a glass petri dish filled with paraffin oil and with the bottom covered by a layer of Sylgard. Immersed in paraffin oil, muscle was manually dissected into small bundles of fibers. Single fibers were isolated from the small bundles and the sarcolemma was mechanically removed with forceps. Fibers were cut at up to 1 cm in length and knots were tied on both ends of the skinned fiber using 10-0 nylon or silk suture, which enabled proper handling of the single fiber with forceps (Fine science tools, Dumont #5) and also served to mount the skinned fiber in a custom built experimental chamber. After skinning, fibers isolated in the paraffin oil filled petri dish were plotted dry on Whatman filter paper and transferred to the experimental chamber filled with internal solution (see below). The chamber had a diameter of about 1.5 cm and was based on top of a 1.5 coverslip. Two parallel Minutien Pins (Fine science tools) glued down onto the coverslip served to hold the fiber by clamping down the suture knot tied at the fiber ends. Once the fiber was fixed in the chamber the preparation was immediately mounted onto the stage of an inverted microscope (Nikon Eclipse Ti-E). The whole procedure, from sacrificing the animal (*t* = 0 min), to the excision of the EDL muscle (*t* = 15 min), the isolation and skinning of single fibers (*t* = 35 min), a possible incubation with Rhod-5N (*t* = 50 min), and the mounting of the fiber within the experimental chamber (*t* = 60 min), allocates a total time of about 1 h, with approximated time points given in brackets. Due to experiments were performed in a sequential manner, subsequent fibers were isolated only after confocal imaging on the preceding fiber was finished, with respective additive temporal delays. To improve preparation quality, room temperature was constantly maintained at 22 degrees and muscle, which was not used immediately, was remained immersed in paraffin oil and kept on ice. Under these conditions successful EFS can be obtained several hours after excision of the muscle. Once mounted into the recording chamber, individual skinned fibers loaded with rhod-5N and fluo-4 that respond to EFS were typically recorded over a time span of several minutes, but we haven’t investigated how stable the preparation is over longer time periods.

To load the t-system with rhod-5N, small bundles of fibers were dissected with fine forceps under paraffin oil from the EDL muscle. Before skinning, a small bundle was exposed to a single drop of Ringer solution containing 2.5 mM of the Ca^2+^-sensitive dye rhod-5N applied by a micro capillary. The drop was left on the fiber bundle for at least 10 min before single fibers were mechanically isolated and skinned. By skinning the fiber the t-system re-seals and traps rhod-5N within its lumen (Lamb et al., 1995; Cully et al., 2016).

### Voluntary Running Wheel Training

Mice were kept in cages equipped with running wheels (ACT-551-MS-SS, Coulbourn Instruments). Mouse running activity was monitored by counting wheel rotations by using a magnetic switch, which was connected to a breakout box (ACT-553, Coulbourn Instruments) through a pair of banana jacks. The breakout box was further connected to an interface (ACT-556a, Coulbourn), which communicated via USB with the recording software (Clocklab; ACT-500, Coulbourn). Recorded data were read and analyzed using the ClockLab analysis toolbox in MATLAB R2015a and Microsoft Excel.

### EFS-Score

We defined the EFS-score as an overall measure of how well fibers isolated from one animal preparation could be electrically stimulated. The EFS-score takes integer values ranging from 0 to 4 based on the following criteria: 0, defines fibers that could not be stimulated at all; 1, only very small fiber segments responded, or small segments with weak Ca^2+^-release; 2, half or less of the fiber responding to EFS with weak Ca^2+^ release; 3, almost complete excitation along the whole fiber length with strong Ca^2+^ release, or excitation along the entire fiber but with weaker release of Ca^2+^; 4, a strong release of Ca^2+^ along the whole fiber. Respective EFS-score levels were assigned if at least one fiber from one animal preparation met the required criterion. The intensity of Ca^2+^ release was determined from the fluo-4 peak amplitude upon stimulation with a cut off at *F*/*F*_0_ = 1.5; weak Ca^2+^ release defined being lower and strong Ca^2+^ release defined being larger, respectively.

### Experimental Solutions

Skinned fibers were bathed in an internal salt solution detailed in previous work (Cully et al., 2016; Koenig et al., 2018). It consisted of (in mM): 90 HEPES, 10 EGTA, 40 HDTA (1,6-diaminohexane-N,N,N′,N′-tetraacetic acid), 8.77 MgO, 1.94 CaCO3, 8 Na2ATP, 10 Na2CP (creatine phosphate). pH was adjusted to 7.1 ± 0.1 with KOH. Free cytosolic Ca^2+^ concentration was calculated to 67 nM assuming a pre-determined *K*_d_ of EGTA for Ca^2+^ of 200 nM (Cully et al., 2016). Free cytosolic Mg^2+^ concentration was 1 mM. BTS (4-methyl-*N*-(phenylmethyl)benzenesulfonamide; #1870, Tocris Bio-Techne) was dissolved in dimethyl sulfoxide (DMSO) and added at a final concentration of 50 μM to inhibit fiber contraction during EFS. Fluo-4 (F14200, ThermoFisher) was dissolved in DMSO at a stock concentration of 5 mM, stored in aliquots, and was added at a final concentration of 10 μM. All solutions were made up freshly on the day of experiment.

### Calibration of fluo-4 and rhod-5N Fluorescence

*K*_d___Ca_ values for fluo-4 and rhod-5N, 1 μM and 0.872 mM, respectively, were taken from previously published work on rat EDL skinned fibers (Koenig et al., 2018). Fluorescence values of fluo-4 and rhod-5N were converted to [Ca^2+^]_cyto_ and [Ca^2+^]_t–sys_ using the following formula for single wavelength dyes assuming quasi steady-state conditions of Ca^2+^-binding to the fluorophore for every acquired image within the obtained image series (Grynkiewicz et al., 1985; Royer et al., 2008; Cully et al., 2016),

(1) $$\left[{\text{Ca}}^{2+}\right]=K_{\text{d}}*\left(F-F_{\min}\right)\,/\,\left(F_{\max}-F\right),$$

with [Ca^2+^] is free Ca^2+^ concentration, *K*_d_ is fluorophore dissociation constant for Ca^2+^, *F* is fluorophore fluorescence, *F*_*min*_ and *F*_*max*_ is fluorophore fluorescence under Ca^2+^ free or high Ca^2+^ conditions in the presence of the ionophores ionomycin (25 μM) and calcimycin (A23187, 25 μM) (Cully et al., 2016). Calibration solution for *F*_*max*_ contained (in mM): 5 CaCl_2_, 140 NaCl, 1 MgCl_2_, 10 HEPES, 4 KCl, 5 glucose, pH 7.4 with NaOH/HCl, with added ionophores and 100 μM BTS. *F*_*min*_ was Ca^2+^ free and contained: 90 HEPES, 10 EGTA, 40 HDTA, 10.3 MgO, 1.94 CaCO_3_, 8 Na_2_ATP, 10 Na_2_CP. pH was adjusted to 7.1 ± 0.1 with KOH; with added ionophores and 50 μM BTS.

To determine [Ca^2+^]_t–sys_ (*t*), Eq. (1) was used, which required determining *F*_*min*_ and *F*_*max*_ for each fiber. *F*_*min*_ was determined at the end of each experiment. *F*_*max*_ was determined by rearranging Eq. (1):

$$F_{\max}=F+\left(F-F_{\min}\right)*K_{\text{d}}\,/\,\left[{\text{Ca}}^{2+}\right],$$

with *F*, the fluorescence before EFS, and [Ca^2+^], the respective free Ca^2+^ concentration in the cytoplasm (67 nM) and the t-system (calibrated in an independent set of experiments), respectively. Independent calibration of the t-system was necessary because exposure of the skinned fibers to the *F*_*max*_ solutions frequently induced the formation of vacuoles, mostly in the longitudinal t-system, which could not be identified on the recordings from the resonant scanner due to its poor spatial resolution. Moreover, due to the high frequency sampling, rhod-5N fluorescence bleaches over time, roughly by about 20% during 2 min of recording, which would induce a large calibration error. Therefore, we performed *n* = 5 experiment using low speed recordings with high spatial resolution that allowed us to identify vacuoles and to exclude these regions from the calibration process. In this way we calibrated [Ca^2+^]_t–sys_ before EFS in the presence of 67 nM [Ca^2+^]_cyto_ and confirmed it to be 1.4 mM in mouse; as determined previously in rat EDL muscle fibers (Cully et al., 2016).

### Electrical Field Stimulation

Electrical field stimulation was applied via a pair of platinum electrodes placed in parallel to the long axis and on opposing sides of a skinned fiber set about 1 cm apart. Monophasic square voltage pulses with amplitudes of 60 V and durations of 2–4 ms were generated by a GRASS S48 square pulse stimulator box and delivered at a frequency of 1, 2, 5, or 10 Hz.

### Confocal Imaging

The fibers were imaged as previously described (Koenig et al., 2019), using a 20× water (Nikon, CFI APO 20× WI λS, long working distance, NA 0.95, or Plan Apo λ 20× NA 0.75) immersion objective on a Nikon laser scanning confocal microscope system (Nikon A1R+ system on an inverted Nikon Ti-E microscope) equipped with a 12 kHz resonant scanner and high-sensitivity GaAsP detectors. Rhod-5N and fluo-4 were excited at 488 and 561 nm at a laser power of 0.4–1% and 0.4–0.8%, and emitted light was collected at 525/50 nm and 595/50 nm, respectively. The pinhole was set to 4–7 Airy units. Acquired image series (xyt) had a physical dimension of *x* = 512 pixel and *y* = 32–128 pixel, resulting in a temporal resolution of Δ*t* = 4–16 ms, or 250–62 frames s^–1^. Fluorescence was averaged across a region of interest to improve the signal to noise ratio, in particular necessary to monitor rhod-5N fluorescence in the t-system.

### Statistical Analysis and Data Fitting

Throughout the manuscript data are presented as means ± SEM. The number of mice used in this study is indicated by *N*, while the number of fibers is denoted by n.

Mean data of the rate constants and [Ca^2+^]_t–sys_ in Figure 5 were fit with single exponential functions of the form, *y* = *y*0 + (*Plateau*−*y*0)*(1−*exp*⁡(−k*x)), were y represents the ordinate values, rate and [Ca^2+^]_t–sys_, respectively, and *x* the abscissa value frequency. *y*0 denotes the initial value at zero frequency, which were constrained to 0 and 1.4 for rate and [Ca^2+^]_t–sys_, respectively, reflecting no depletion and a constant level of [Ca^2+^]_t–sys_ if no EFS was applied. Plateau reflects the *y* value in the limit of infinite frequency, and *k* the exponential rate constant. To compare data from mouse with those derived from rat, respective fits were compared with an Extra sum-of-square *F*-test. A *p* value <0.05 was considered significant.

### Rights and Permissions

Data from rat EDL muscle fibers were taken from Koenig et al. (2018) published under CreativeCommons BY 4.0 license.

## Results

**FIGURE 1:**
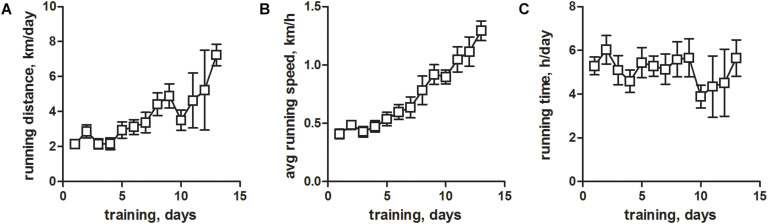
Voluntary running wheel parameters of housed mice. Male C57BL/6 mice at an age of 10–15 weeks were trained between 1 and 14 days by providing a running wheel in their cages. Training parameters of voluntary wheel running were monitored continuously for individual animals. **(A)** Total daily running distance (kilometers per day), **(B)** average daily running speed (kilometers per hour), and **(C)** total daily running time (hours per day). All values are given as means ± SEM and are derived from *N* = 29 mice.

In rat, EFS can trigger APs in the sealed t-system and subsequent Ca^2+^ release from the SR in skinned, fast-twitch EDL muscle fibers (Posterino et al., 2000; Posterino and Lamb, 2003; Goodman et al., 2008; Launikonis et al., 2009; Koenig et al., 2018, 2019). In contrast, EFS works much inferior in skinned fibers from rat and mouse slow-twitch soleus muscle and, importantly, in skinned fibers from mouse EDL muscle. The reason for that is unknown. The inability to use mouse mechanically skinned muscle fibers for EFS represents an impediment to research efforts. We suspected that the inability of mouse skinned skeletal muscle fibers to respond to EFS arose from a significant physical inactivity of experimental mice housed in standard cages, which substantially restrains their natural running behavior. To overcome this, we provided the mice with voluntary running wheels for 1 to 14 days and tested the excitability in mechanically skinned fibers prepared from the EDL muscles thereafter. Continuous monitoring of the physical activity revealed a status of significant de-training in conventionally housed mice (Figure 1A–C). Thus, at the beginning of training, mice ran a distance of about ∼2 km/day (Figure 1A) at an average speed of ∼0.5 km/h (Figure 1B). These values are considerably lower than commonly published values, where mice typically travel distances of 4 km/day at an average speed of about 1 km/h (Coleman et al., 1998; Manzanares et al., 2019). Respective activity values increased considerably with continuous training times (Figures 1A,B) confirming our hypothesis that mice housed in standard cages show signs of substantial de-training. Running times on the other hand did not change during the whole training period (Figure 1C), and recorded values matched previously published ones (Coleman et al., 1998; Manzanares et al., 2019).

### Electrical Field Stimulation Induces SR Ca^2+^ Release in Skeletal Muscle Fibers of Trained Mice

**FIGURE 2:**
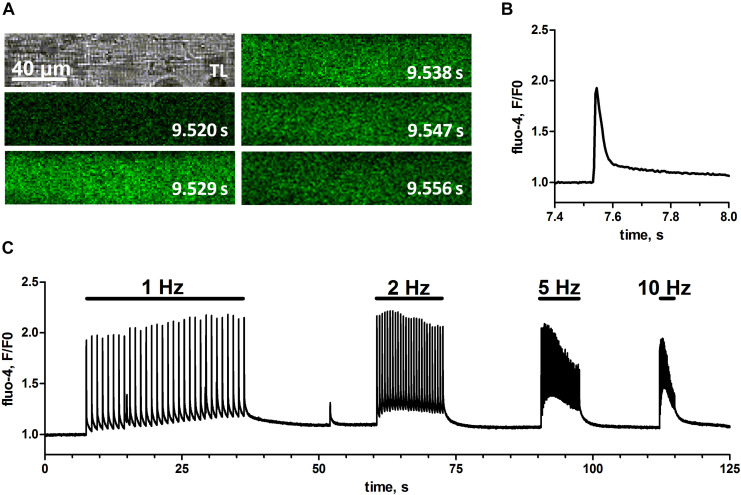
Cytosolic Ca^2+^-transients upon electrical field stimulation in skinned mouse skeletal muscle fibers. EFS was applied via two platinum electrodes in parallel to the fiber’s long-axis by rectangular monophasic voltage steps of 60 V with 4 ms duration. A skinned mouse EDL muscle fiber was bathed in a solution containing endogenous free Ca^2+^, Mg^2+^, and ATP concentrations. **(A)** Original transmitted light image of the skinned fiber (top left) and respective image series of fluo-4 fluorescence with indicated time stamps referring to the time course of single EFS pulse induced Ca^2+^-transient (left to right, top to bottom). Scale bar refers to *x*- and *y*-dimension. Note that, in order to obtain a maximal imaging speed, recorded image series were restricted to a narrow rectangular region of interest within the fiber; therefore no area surrounding the fiber can be seen. **(B)** Profile of the fluo-4 fluorescence plotted as fluorescence over baseline fluorescence (*F*/*F*_0_) during the first delivered EFS pulse. **(C)** Profile of fluo-4 fluorescence (*F*/*F*_0_) during trains of EFS delivered at different frequencies of 1, 2, 5, and 10 Hz. Original image series and respective fluo-4 profile were obtained from one representative fiber.

To test if the physical training of mice would result in skinned muscle fibers that respond to EFS we isolated single fibers of EDL muscle derived from animals that had used a voluntary running wheel (Figure 1). These fibers were mechanically skinned by removing the sarcolemma with forceps, which causes the t-system to seal and leaves the fiber’s cytoplasm accessible to the surrounding bath solution but preserves the fiber structure and function otherwise (Lamb and Stephenson, 1994; Dutka et al., 2008; Cully et al., 2017). Subsequently, these skinned fibers were immersed in a physiological salt solution mimicking the cytoplasmic environment of the cell, with free Ca^2+^ strongly buffered by 10 mM EGTA. The added Ca^2+^-sensitive dye fluo-4 allowed for real-time imaging of changes in the cytosolic free calcium concentration with confocal microscopy, while EFS was applied by a pair of platinum electrodes placed in parallel to the longitudinal axis of the fiber (Posterino et al., 2000; Koenig et al., 2018, 2019). Figure 2, shows one of the first successful EFS in a skinned EDL fiber from a trained mouse. Individual electrical field pulses elicited clearly discernible transient rises in fluo-4 fluorescence indicating a respective increase in free cytosolic Ca^2+^ ([Ca^2+^]_cyto_) due to SR Ca^2+^ release. The placing of the stimulating electrodes in parallel to the fiber’s long axis led to an induction of APs in the sealed t-system (Posterino et al., 2000) simultaneously across sarcomeres, as evidenced by a uniform excitation pattern across the whole fiber length (Figure 2A). The high EGTA buffering in the cytosol led to Ca^2+^-transients of brief duration and with only a small increase in baseline fluorescence after release (Figure 2B). The fiber could successfully be stimulated at frequencies of 1, 2, 5, and 10 Hz with the higher frequencies leading to an increase of baseline Ca^2+^ levels during trains of stimulation (Figure 2C).

### Moderate but Not Excessive Exercise Enables Successful Electrical Field Stimulation of Mouse Skinned Skeletal Muscle Fibers

**FIGURE 3:**
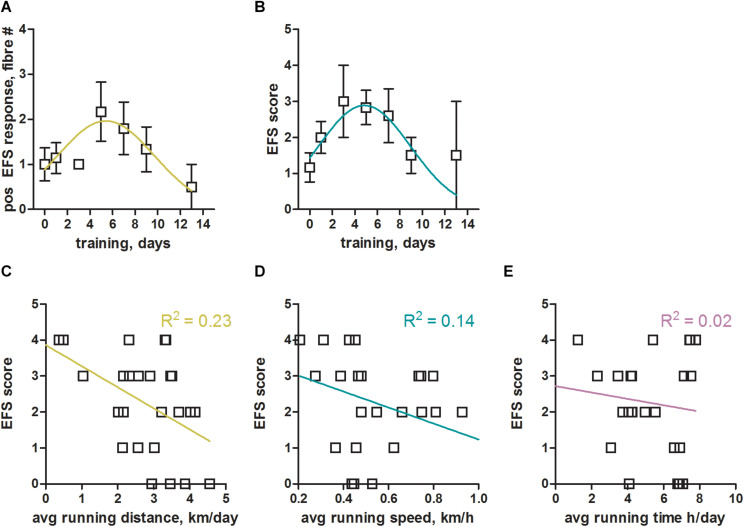
Optimal training parameters for successful electrical field stimulation of mouse skeletal muscle fibers. **(A)** Total number of mouse EDL fibers from one experiment day (one animal) with positive response to EFS plotted over the number of training days (voluntary wheel running). Data were binned into two day intervals, [1–2], [3–4], [5–6], [7–8], [9–10], [11–12], and [13–14], with the number of mice in these intervals being 7, 3, 6, 5, 6, 0, 2, respectively. Data from *N* = 6 non-trained mice (no running wheel) were included as control at 0 training days. A Gaussian fit to the binned data revealed a maximum (mean ± SEM) at 5.4 ± 0.9 days of training; The Gaussian fit was the preferred model when compared to a straight line (*p* < 0.05, Extra sum-of-squares *F*-test). On average we tested 5 fibers per animal, so that a value of 2 would indicate that 40% of fibers showed a positive response to EFS. **(B)** EFS-score, a measure of the quality of EFS induced SR Ca^2+^ release (see section “Materials and Methods” for details) estimated from all fibers derived from one animal, plotted over the number of training days. Data were binned into two day intervals as in **(A)**. A Gaussian fit to the binned data revealed a maximum (mean ± SEM) at 4.9 ± 0.6 days of training. The Gaussian fit was the preferred model when compared to a straight line (*p* < 0.05, Extra sum-of-squares *F*-test). **(C–E)** EFS-score plotted over the average running distance, speed, and time of respective mice. Linear regression lines with respective *R*^2^ values are shown in color. Data were derived from *N* = 29 trained and *N* = 6 non-trained mice.

Without training we were consistently struggling to obtain robust responses to EFS in mouse EDL muscle fibers. However, already a few days of voluntary wheel running led to a convincing stimulation of mouse EDL skinned fibers (Figure 2). In order to systematically derive optimal training conditions, we sacrificed animals that had been trained for between 1 and 14 days. We assessed the benefit of training by counting the number of fibers within one preparation (one animal per experiment day) that responded positively to EFS (Figure 3A). As a second readout, we defined a specific score (EFS-score; see section “Materials and Methods” for definition details), that provided a measure to estimate the overall EFS quality of individual preparations (Figure 3B). Both parameters indicated similar training times for an optimal response to EFS, peaking between 5 to 6 days of training (Figures 3A,B). Surprisingly, longer training periods did not further improve the response to EFS, but led to a reduction in fiber excitability. Taken together, voluntary running on a wheel for 5 to 6 days not only led to an approximately two-fold improvement of the number of EFS positive fibers (Figure 3A) but also increased the quality of respective EFS responses by almost three-fold (Figure 3B).

We next analyzed which of the training parameters (see Figure 1) would have the strongest impact on the obtained results. To this end we plotted the EFS-score over the running distance, running speed, and running time (Figures 3C–E) to derive potential correlations. The EFS-score correlated weakly in a negative fashion with the average running distance (Figure 3C) and running speed (Figure 3D) of mice, indicating that fibers from mice that exercised more intensely were less likely to respond to EFS. No correlation was observed for the EFS-score with the average running time of the mice (Figure 3E).

### Skeletal Muscle Fibers From Trained Mice Show Phasic SOCE

**FIGURE 4:**
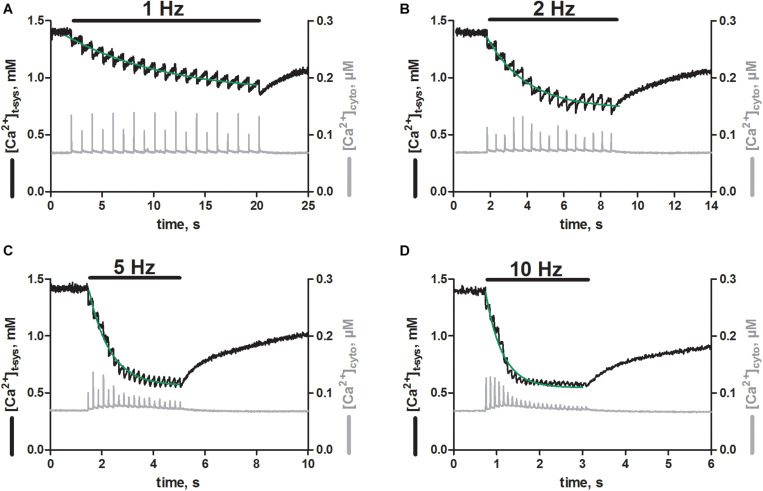
Skeletal muscle fibers from trained mice show phasic SOCE upon electrical field stimulation. Typical recordings of [Ca^2+^]_t–sys_ (black traces, left axes) and [Ca^2+^]_cyto_ (gray traces, right axes) over time as derived from skinned mouse EDL fibers during EFS. xyt image series were acquired simultaneously for the fluorescent signals of rhod-5N and fluo-4 trapped in the t-system and loaded into the cytosol, respectively. Both signals were spatially averaged over a region of interest, in which the fiber was responding optimal to EFS. Signals were further calibrated by determining the fluorescent values under conditions of minimal and maximal Ca^2+^ concentrations in the presence of ionophores (see section “Materials and Methods”). Electrical stimulation at 1 **(A)**, 2 **(B)**, 5 **(C)**, and 10 Hz **(D)** as indicated by the black bars was started shortly after beginning of the recording and was continued until a new steady state in the t-system was reached. Of note, sampling below the Nyquist criterion resulted in an apparent modulation of cytosolic Ca^2+^ transient amplitudes, best seen in A. Green lines represent mono-exponential fits to the decaying phase of [Ca^2+^]_t–sys_ upon stimulation. Original profiles of [Ca^2+^]_t–system_ and[Ca^2+^]_cyto_ were obtained from one representative fiber.

Next, we were interested if the EFS triggered SR Ca^2+^ release was associated with activation of pSOCE, as we previously observed in rat EDL skinned fibers (Koenig et al., 2018, 2019). To this end we loaded the t-system and the cytoplasm of EDL muscle fibers derived from trained mice with the Ca^2+^-sensitive dyes, rhod-5N and fluo-4, respectively (Cully et al., 2016; Koenig et al., 2018, 2019). The dual loading allowed us to track the Ca^2+^ movements in both of these compartments simultaneously, while triggering Ca^2+^ release from the SR. Thus, EFS triggered SR Ca^2+^-release with each stimulation pulse applied (Figure 4, gray traces), as observed originally in fibers loaded with fluo-4 only (Figure 2). In addition, each stimulation pulse resulted in an abrupt depletion of free Ca^2+^ in the t-system ([Ca^2+^]_t–sys_; Figure 4 black traces). Repeated stimulation at a frequency of 1 Hz (Figure 4A, black bar) led to a step-wise depletion of steady-state [Ca^2+^]_t–sys_ with respect to the level before the start of EFS. The loss of [Ca^2+^]_t–sys_ upon individual EFS pulses was partially recovered by a re-uptake of Ca^2+^ into the t-system in between stimulation pulses, which is most likely carried by the sodium-calcium exchanger (Cully et al., 2018). The single depletion and re-uptake steps generated a case like depletion pattern in [Ca^2+^]_t–sys_, which, dependent on pulse frequency, converged toward a new steady-state level with prolonged stimulation. The dynamic equilibrium was reached faster at higher stimulation frequencies of 2, 5 and 10 Hz compared to 1 Hz, and led to a deeper overall depletion of the [Ca^2+^]_t–sys_ during continuous EFS (Figures 4B–D). Overall, the EFS-induced staircase like depletion pattern appeared very similar to what we had previously described in rat EDL skinned muscle fibers (Koenig et al., 2018, 2019).

**FIGURE 5:**
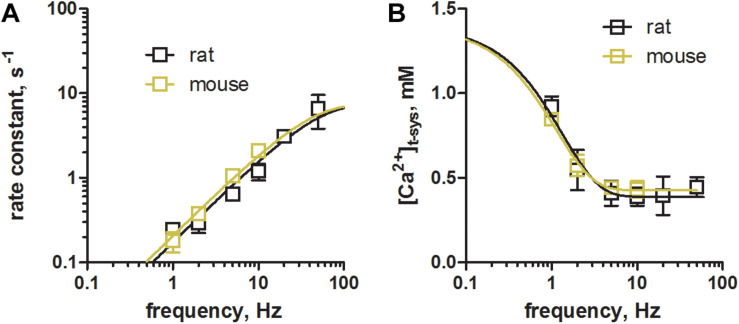
Comparison of phasic SOCE between mouse and rat skeletal muscle fibers. Skinned EDL muscle fibers were derived from rats and trained mice using identical experimental protocols. **(A)** Comparison of the rate constants during a train of EFS at different frequencies as shown in Figure 4. Rate constants were derived from mono-exponential fits to the overall staircase like depletion pattern of [Ca^2+^]_t–sys_ (green fits, Figure 4). Mean ± SEM data derived from mouse fibers (*N* = 3 animals) as obtained within this study are shown in olive green, while data derived from rat fibers (*N* = 5 animals, in black), were taken from our previous work (Koenig et al., 2018, CC BY 4.0 license). **(B)** Comparison of the new steady-state [Ca^2+^]_t–sys_ levels during repeated stimulation as determined by the dynamic equilibrium between depletion and reuptake fluxes. Data from mouse (olive green) and rat (black) EDL muscle fibers are derived from the same sources as in **(A)**. Exponential fits to the data derived in mice and rats were compared using an Extra sum-of-square *F*-test. Calculated p values of 0.26 and 0.89 for the rate constant and for [Ca^2+^]_t–sys_, respectively, suggested the preferred model to have the same fit parameters for the two data sets.

### Phasic SOCE Is Similar in Rat and Mouse EDL Fibers

Next, we compared the characteristics of pSOCE in mouse EDL skinned fibres (the present study) to those previously established in rat EDL skinned fibres (Koenig et al., 2018). Figure 5 shows a comparison of the rate of depletion and the new steady-state levels of [Ca^2+^]_t–sys_ induced by pSOCE during trains of EFS in mouse and rat fibers. Respective values were derived by fitting the staircase-like depletion pattern with a single exponential function to obtain the rate and steady-state values for different stimulation frequencies (1, 2, 5, and 10 Hz). Mean ± SEM values from a total of *n* = 3 mouse EDL fibers (Figures 5A,B, in olive green), together with the respective data from *n* = 10 rat EDL fibers (in black; taken from Koenig et al., 2018 with permission), were displayed on top of each other for comparison. Overall it can be observed that rat and mouse show very similar characteristics. These results suggest that phasic SOCE is a common mechanism in mammalian skeletal muscle, occurring with similar kinetics.

## Discussion

Within this work we have relied on the assumption that mouse limb muscle is de-trained when animals are housed in cages not supporting their natural running behavior and that this can be overcome by providing running wheels. While this approach clearly led to efficient EFS of mouse EDL skinned fibers, in a manner comparable to previous experiments in rat EDL skinned fibers, our study cannot provide a respective rational explanation. Thus, we can only speculate about possible underlying reasons.

Voluntary wheel running affects fiber type composition (Wernig et al., 1990; Allen et al., 2001), whereby a typical effect of endurance training in fast-twitch muscle is a relative switch to more oxidative fiber types. This conversion could render the fibers more susceptible to EFS. This, however, seems unlikely because of two points. First, we have consistently failed to obtain successful EFS in skinned fibres from mouse and rat soleus muscles, which express almost exclusively slow-twitch fibers. Second, training of 5 to 6 days only, the optimal training period determined in Figure 3, is likely insufficient to allow for a considerable fiber type switch, given that respective adaptations take several weeks to occur (Wernig et al., 1990; Allen et al., 2001).

An alternative explanation why EFS improves in moderately exercised mouse muscle might be associated with molecular changes underlying the excitability of the fiber. (i) EFS works equally well in intact (e.g., Fryer and Neering, 1989) and skinned rat EDL fibers (Posterino et al., 2000; Koenig et al., 2018, 2019). Accordingly, VGSCs within the t-system and not the sarcolemma have been identified as respective EFS response elements (Posterino et al., 2000). VGSCs cluster at the entry points of the t-system (Murphy et al., 2009) and protrude into the sub-sarcolemma region (Clausen, 2003). It is thus conceivable that, while some of these channels are removed during the skinning procedure, a significant fraction of channels remains functional in sub-sarcolemma regions of the fiber. Upon exercise, an increased AP upstroke was observed in rat soleus fibers, suggesting a larger density of VGSCs or an increased fraction of non-refractory channels (Broch-Lips et al., 2011). If this holds true for fast twitch muscle and for shorter training periods then it might serve as an explanation for why EDL muscle fibers of trained mice do successfully respond to EFS. (ii) Fibers from trained animals were more resistant to depolarizations by high K^+^ indicating a more robust t-system membrane potential as caused, e.g., by an increase in Na^+^/K^+^ pump activity (Clausen, 2003; Broch-Lips et al., 2011). Indeed, an increased Na^+^/K^+^-ATPase content was detected in muscle fibers derived from trained compared to sedentary rats (Xu et al., 2018). (iii) Training induced a lower resting conductance, possibly carried by Cl^–^ (Broch-Lips et al., 2011), which is in line with an observed increased Cl^–^ conductance in sedentary muscle (Pierno et al., 2007). Importantly, a reduction in resting Cl^–^ currents was shown to improve fiber excitability (Coonan and Lamb, 1998; Pedersen et al., 2005). While it is unclear if these adaptions occur already after a few days of voluntary wheel running, it is conceivable that the aforementioned and possibly additional unknown factors might contribute to the observed increase in excitability of mouse EDL skinned fibers upon moderate training.

Moderate exercise on a running wheel for 5–6 days led to a significant improvement of the number of EFS positive fibers (Figure 3A) as well as the quality of respective EFS responses (Figure 3B). Thus, 5–6 days of moderate excise in mice offers an about six-fold improvement in EFS, making EC coupling and dual EC coupling - pSOCE experiments feasible in mouse skinned fibres. A somewhat paradoxical finding was that training times longer than 5–6 days led to an apparent decline in fiber excitability (Figures 3A,B). The reasons for that are unknown. Training times, frequency and performance differ substantially among individual mice on voluntary running wheels. A more standardized approach, e.g. using a treadmill, may help define such parameters in EFS success.

### Physiological Relevance

Chronic SOCE (cSOCE), as induced by thorough depletion of the SR Ca^2+^-stores, has been observed in skeletal muscle across mammalian species (Kurebayashi and Ogawa, 2001; Stiber et al., 2008; Cully et al., 2016), including human (Cully et al., 2018), and there is little doubt that cSOCE is carried by STIM1 and Orai1 proteins (Lyfenko and Dirksen, 2008; Stiber et al., 2008; Dirksen, 2009). Phasic SOCE (pSOCE), on the other hand, as activated by individual APs during EC-coupling (Koenig et al., 2018, 2019), was only observed in rat EDL muscle fibers to date, and its molecular nature is still unresolved. Although it seems reasonable to assume that pSOCE is mediated by the same protein machinery (Koenig et al., 2018), a direct proof of this hypothesis remains outstanding.

To provide an explanation for the extraordinary fast activation of pSOCE (Launikonis and Ríos, 2007; Edwards et al., 2010; Koenig et al., 2018, 2019) a direct and permanent physical coupling of STIM1 and ORAI1 has been proposed (Edwards et al., 2010; Launikonis et al., 2010) in combination with a nanodomain Ca^2+^ depletion within the terminal cisternae of the SR (Koenig et al., 2019) upon opening of the ryanodine receptor during EC-coupling. This notion was supported by respective co-localization of STIM1 and ORAI1 at the triad membranes (Darbellay et al., 2011; Wei-Lapierre et al., 2013; Tammineni et al., 2020), and encouraged by the discovery of a long STIM1 isoform (STIM1L) with supplemental actin binding properties and the ability to form permanent STIM1L/ORAI1-clusters (Darbellay et al., 2011; Saüc et al., 2015). However, others have questioned that idea based on results derived from an (uncalibrated) STIM1/ORAI1 complementation assay (Wei-Lapierre et al., 2013).

Store-operated calcium entry plays an important role in muscle growth and development, is involved in fatigue resistance, and causes different forms of myopathy when impaired (Feske, 2009). The underlying mechanisms are poorly understood. Partly, this might be caused by the fact that chronic, long-lasting activation of SOCE does not reflect the natural activation patterns of skeletal muscle, which occur as brief twitches induced by very transient releases of SR Ca^2+^. The temporal presentation of SOCE in working, intact skeletal muscle should not be assumed from experimental conditions where SOCE and SR Ca^2+^ release are not simultaneous measured or there is SR Ca^2+^ depletion due to inhibition of the SR Ca^2+^ pump or removal of extracellular Ca^2+^. Also, an increase in persistent Ca^2+^ leak through the ryanodine receptor is required to activate cSOCE (Cully et al., 2018), which is more likely to be associated with disease conditions (Cully et al., 2018; Rebbeck et al., 2020) or the post-exercise period (Place et al., 2015; Ivarsson et al., 2019). In this light it seems reasonable to assume that pSOCE rather than cSOCE actually represents the more relevant physiological manifestation of this important Ca^2+^ flux in prevailing skeletal muscle function.

Mouse models, genetically engineered to target the proposed key proteins of SOCE, have already been generated, but the inability to efficiently use mechanically skinned muscle fibers from mouse for EFS has presented significant impediment to research efforts. With the here presented protocol of moderate exercise, providing a running wheel to otherwise physically restrained mice, we have provided a tool to measure pSOCE in mouse muscle during single muscle twitches. Genetically modified mouse models can now be put forward to test the molecular nature and physiological role of pSOCE. Furthermore, a new platform is provided to study this sophisticated type of Ca^2+^-influx in skeletal myopathies, in which pSOCE presumable plays a prominent role.

### pSOCE in Intact and Skinned Fibers

Phasic SOCE has been demonstrated directly in skinned fibers (Koenig et al., 2018). The skinned fiber preparation provides means to simultaneously measure t-system Ca^2+^ influx and SR Ca^2+^ release during EC coupling with a high signal: noise ratio and temporal resolution for detecting Ca^2+^ entry (Launikonis and Ríos, 2007; Koenig et al., 2018, 2019). In intact fiber preparations, resolving brief Ca^2+^ entry is difficult (Launikonis et al., 2009). However, there is evidence from indirect measurements in intact fiber preparations of a “pSOCE” (Bianchi and Shanes, 1959; Curtis, 1966; Gissel and Clausen, 1999). For example, Gissel and Clausen (1999) showed that a continuous, low-frequency EFS of intact muscle for several hours led to a significant influx and accumulation of radioactive Ca^2+^ consistent with stimulation frequency that was resistant to nifedipine. Studies of SOCE in intact fibers with high frequency EFS have suggested SOCE is involved in the continuing Ca^2+^ release response. The indirect nature of the measure of SOCE in these studies (no measure of Ca^2+^ entry during EC coupling) means we need to apply what we have measured in skinned fibers to help determine what is happening in the intact system. The use of genetically manipulated mice in skinned fiber EFS experiments, using the protocols developed here, will provide a more solid platform to tie results from intact and skinned fibers together, and advance the field.

## Data Availability Statement

The raw data supporting the conclusions of this article will be made available by the authors, without undue reservation.

## Ethics Statement

The animal study was reviewed and approved by the Animal Welfare Committee at the Medical University of Vienna and is covered by licence BMBWF 2020-0.499.046 granted by the Federal Ministry of the Republic of Austria.

## Author Contributions

XK and BL contributed to the conception and design. EL and XK performed the experiments and analyzed the data. EL and BL contributed reagents, materials, and analysis tools. XK, KH, and BL wrote the manuscript and contributed to the data interpretation and manuscript revision. All authors contributed to the article and approved the submitted version.

## Conflict of Interest

The authors declare that the research was conducted in the absence of any commercial or financial relationships that could be construed as a potential conflict of interest.
